# Mobile application for monitoring patients under home oxygen therapy: a protocol for a randomized controlled trial

**DOI:** 10.1186/s12875-021-01450-8

**Published:** 2021-05-26

**Authors:** Anisbed Naranjo-Rojas, Luis Ángel Perula-de-Torres, Freiser Eceomo Cruz-Mosquera, Guillermo Molina-Recio

**Affiliations:** 1 School of Medicine, GINEYSA - GISI. USC Research Groups, Universidad Santiago de Cali, Cali, Colombia; 2 Multiprofessional teaching unit for Family and Community Care of the Córdoba and Guadalquivir District. Maimonides Biomedical Research Institute of Cordoba (IMIBIC), Hospital Universitario Reina Sofia, Universidad de Córdoba, Cordoba, Spain; 3 Nursing, Pharmacology and Physiotherapy Department, University of Cordoba. Lifestyles, Innovation and Health (GA-16). Maimonides Biomedical Research Institute of Cordoba (IMIBIC), Cordoba, Spain

**Keywords:** Oxygen therapy, COPD, E-Health, Mobile applications, Home healthcare

## Abstract

**Background:**

Mobile technologies have become capable of changing the paradigm of healthcare services. A clear example is that, nowadays, these technologies are an important instrument for data collection processes, epidemiologic surveillance, health promotion and disease prevention. Therefore, technological tools should be exploited to optimize the monitoring of patients with chronic diseases, including patients who require home oxygen therapy. Purpose: The purpose of this study is to determine the efficacy of a mobile application in the clinical monitoring of patients under home oxygen therapy.

**Methods:**

This is a randomized controlled trial includes subjects of 18 years or older diagnosed with Chronic Obstructive Pulmonary Disease (COPD) who are under home oxygen therapy. Subjects will be divided into two arms: the intervention group will include patients who will be monitored with a mobile application, and the control group will include patients monitored by conventional follow-up methods (periodic visits of a respiratory therapist). The following outcome variables will be considered to measure the effect of the intervention: identification of dyspnea self-management, number of acute exacerbations associated with oxygen therapy, and the occurrence of oxygen supply underuse.

**Discussion:**

This study is expected to assess the efficacy of a mobile application in the follow up of patients under home oxygen therapy. It will also determine whether the monitoring of a six-month intervention by a team comprising a physician, a nurse and respiratory therapists can decrease acute exacerbations, determine the most appropriate oxygen dose, and identify the underuse of oxygen systems and supplies.

**Trial registration:**

The study has been registered at ClinicalTrials.gov (NCT04820790; date of registration: March 29, 2021)

## Background

Continuous home oxygen therapy (CHOT) is the continuous, general and/or undefined administration of oxygen in the home of patients with chronic hypoxemia. This therapy is aimed at increasing hypoxemic patients’ life expectancy, raising their tolerance to physical activity and decreasing clinical impairment because of reduced blood oxygen levels [1, 2]. In 2001, an increase in the prevalence of Chronic Obstructive Pulmonary Disease (COPD) was observed in Colombia, along with an increase of home oxygen prescriptions for the first stages of therapy, resulting in 3,176 patients enrolled in a home healthcare program. However, no information about their sociodemographic and epidemiologic characteristics is available, which can be explained by the scarce number of epidemiological studies about this therapy. However, González et al. highlight that current prescriptions should be reviewed, and research should be performed to establish guidelines for recommending home oxygen therapy in patients with COPD [3, 4]. In Colombia, the largest COPD registry was created as a result of the Prepocol study (Prevalence of COPD in 5 Colombian Cities Situated at Low, Medium and High Altitude, published in 2008) [5], in which a prevalence of spirometrically defined COPD of 8.9% was estimated. However, no recent data have been collected in new epidemiological studies. It is worth mentioning that the World Health Organization (WHO) described COPD as the third cause of death [6] worldwide, representing a public health concern and one of the primary morbidity and mortality causes. Regarding the costs of hospitalization and COPD exacerbations in Colombia, each exacerbation costs an average of $98, and this value can reach up to almost $700 during hospitalization [7]. Oxygen therapy is essential for COPD management because it can control symptoms, reduce hospital stays and increase survival [8]. However, adherence to this therapy should be consistently monitored. A study performed by Barruecos et al. [9, 10] reported a high rate of noncompliance of patients on home oxygen therapy. Consequently, Mesquita et al. [11] revealed that the quality of life of COPD patients who fail to adhere to oxygen therapy is lower in the long term than that of patients adhering to such therapy. Exacerbations may be observed in patients with COPD, characterized by dyspnea, increased coughing and discharge changes. Moreover, two or more exacerbations per year are considered as acute exacerbations of the disease and have a negative impact on patients’ health. A significant percentage of COPD patients discharged from hospital after an acute exacerbation are readmitted within the next three months, increasing the emergency services attendance rate. Certain factors associated with readmission include poor in-home care, non-adherence to treatment, and limited information about their condition [8, 11]^.^ Therefore, it is essential to develop in-home follow-up and monitoring strategies because home visits allow for a real-time patient examination in the place where therapy usually takes place and are well-accepted by patients [12, 13]. Although the benefits and the clinical and gasometrical criteria for the use of oxygen supplies are well described in literature, the availability of protocols and registry and follow-up instruments for patients hospitalized at home is significantly lesser [1, 3]. Therefore, new healthcare technologies should be used to monitor these patients at home. E-health incorporates information and communication technologies (ICTs) to healthcare products, services, and processes. In this sense, it is defined as the set of ICTs tools that are used in a healthcare environment for prevention, diagnosis, treatment, and follow-up activities, as well as for health management, improving the efficacy of the latter [4]. This term encompasses different healthcare products and services, such as mobile applications (apps) and telemedicine, which includes diagnosis, treatment, and health education. With this technological resource, it is possible to improve healthcare services, save time and money and simplify access of health professionals to remote areas. Moreover, the term “mHealth” should be mentioned as a part of eHealth. Although no standardized definition has been made for this term, the Global Observatory for eHealth (GOe) defined it as a public health medical practice compatible with mobile and wireless devices, such as smartphones, for monitoring patients and the use of virtual assistants [14]. Because mobile health (mHealth) uses smartphones as a complement to medical healthcare, some of its benefits are the possibility of overcoming certain barriers associated with clinical healthcare and adopting technological and innovative interventions to achieve safer clinical practices through resource optimization and patient and/or caregivers’ active participation [15, 16]. These eHealth platforms are an opportunity to develop research, thus providing additional methods to collect, process and analyze health data. Moreover, certain authors focus on the development of new approaches of citizen participation in science [3, 5]. To summarize, it is remarkable that a health professional, patient and/or caregiver use a smartphone and a specific app to access a data source that can be used by the scientific community for their research, thus improving healthcare processes [17]. For the abovementioned reasons, the use of mobile technologies in healthcare is becoming a reality capable of changing the current healthcare paradigm. App functions include data collection, epidemiologic surveillance, patient follow-up, health promotion, disease prevention, access to health data and emergency management [17]. In any case, it seems evident that technologies can address the necessity of achieving a continuous connection in the control of health-disease processes through ICTs [16]. The most innovative aspects of these technologies include real-time information transmission, the possibility to access management guidelines for clinical decision making, and content customization [6]. In a study conducted by Mirkovic et al. [18], aaimed at assessing the usability of Connect Mobile — an app that granted access to an online system providing support to cancer patients in the management of health problems and involved final users in the application design — highlighted the importance, the requirement, and the potential of integrating mobile phones and tablets into patient healthcare systems, as well as of respecting and considering the design suggestions made by users [18]. In this sense, it would be interesting to approach the clinical monitoring of patients on home oxygen therapy through a mobile app aimed at achieving improvements in the treatment, prescription, information, and safety of patients during in-home healthcare processes. Pereira et al. [19], in collaboration with the School of Health Sciences of Universidad de Averio, Portugal, developed Exercit@rt, an app capable of monitoring COPD patients’ real-time heart rate and oxygen saturation levels through a Bluetooth oximeter. Based on this technology, patients can perform, control, geolocalize and assess different respiratory exercises, as well as common daily physical activities. Moreover, this research project incorporated a validation stage involving ten patients with respiratory disorders: five smartphone’s frequent users (SFU) and five smartphone’s non-frequent users (SNFU). The main results revealed that all participants recognized the usefulness of monitoring their disease using the Exercit@rt mobile app [19]. In a literature review about the effectiveness of eHealth interventions in COPD cases, Hallensleben et al. reported a meta-analysis of 10 studies about the teleassistance of this condition, in which a decrease in the number of visits to the emergency and hospitalization rooms was observed as opposed to the control groups receiving regular care [20, 21]. Rassouli et al. [22] digitalized information about patients attending a pulmonary rehabilitation program through a mobile app, which evidenced changes in patients’ quality of life over a of 20-day exercise period. Moreover, Velardo et al. [23] designed an eHealth platform for COPD patients, which also included a mobile app, such that they could manage and control their condition. Patients used the app to complete a symptoms diary, and their oxygen saturation was measured with a wireless pulse oximeter. The clinical trial evidenced a high compliance of self-control during a prolonged period (12 months). Marscha et al. [24] conducted a controlled, randomized trial with an intervention group and a control group of patients with cardiovascular diseases. They designed the Vascular View software to increase self-management behavior of patients with cardiovascular conditions. The intervention group was granted 12-month access to an online self-management software (Vascular View). Assessments took place at baseline and after 6 and 12 months. The results confirmed positive and significant changes over the 12-month period in the intervention group. Arostegui et al. [25] developed an app (PrEveCOPD) to predict adverse events and complications in the short-term progression of patients with COPD. This app is interesting because it shows how clinical prediction measures can be summarized into simple and user-friendly tools, which can be used to estimate the short-term risk of mortality and admission to ICUs. To summarize, it seems evident that health mobile technologies are providing different strategies for the long-term management of chronic diseases, in addition to being convenient, cheap and interactive [26]. Moreover, Bitsaki et al. suggest that technological health tools aimed at chronic patients are a new, sustainable, and innovative business model resulting in significant cost reductions for the health system [27]. Furthermore, the advantages of using technological tools, such as health mobile apps, are that they increase connectivity, reduce the amount of time spent in decision-making, and optimize the use of resources aimed at home healthcare [28, 29]. However, despite the aforementioned positive experiences in the use of mobile apps for different groups of chronic patients, the number of Latin American and Colombian apps is lesser, and this issue should be addressed since most home oxygen supplies are usually underused by patients, which results in an increase of Health System costs and a decrease in coverage based on the requirement for oxygen cylinders. This could be avoided if technological tools with adequate monitoring were incorporated. Thus, we propose this protocol whose purpose is to assess the efficacy of a mobile app in the monitoring of patients under home oxygen therapy through an intervention aimed at decreasing acute exacerbations and increasing self-management of oxygen supplies at patients’ home.

## Methods/design

### Study design

**Fig. 1:**
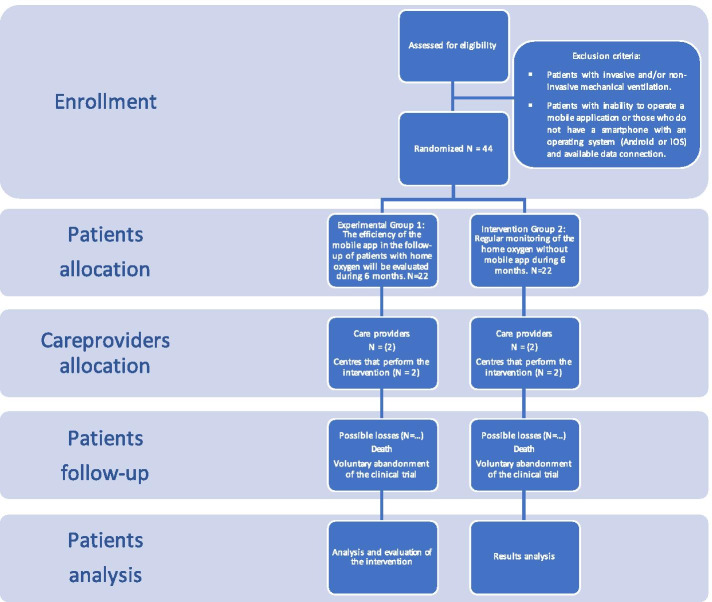
Modified CONSORT flow diagram for individual randomized controlled trials of nonpharmacologic treatments

A randomized, controlled, single-blind, two-arm parallel clinical trial will be conducted during six months to assess the efficacy of a mobile application in the follow-up of patients on home oxygen therapy. The control group will include patients on oxygen therapy subject to conventional follow-up methods (regular visits of a respiratory therapist), while the intervention group will include patients followed through conventional methods and through the use of the mobile app “AppO_2_” (Fig. 1).

### Sample size estimation

A total of 32 subjects (16 for the experimental group and 16 for the control group) should be recruited to obtain a 1:1 ratio, 80% power (beta error = 20%) and 95% safety (alpha error = 5%), and assuming according to the results evidenced by Nguyen et al. [28] that intervention in the experimental group will cause a 3% increase, with ± 6 standard deviation, in the identification of dyspnea self-management. A final sample size of 44 subjects (22 for the experimental group and 22 for the control group) has been determined to minimize the effects of potential losses in statistical power.

### Cluster-randomised trial intervention scheme

#### Recruitment

**Fig. 2:**
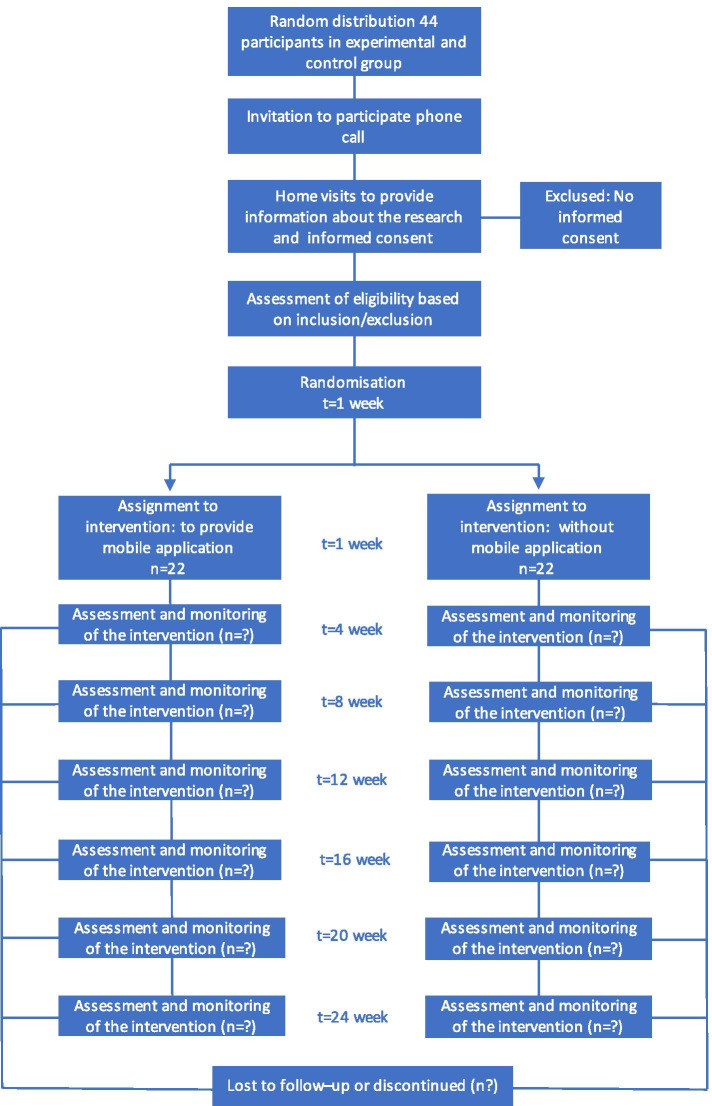
Participant Timeline

Where participants will be recruited: home care servicesTodomed, by whom: respiratory therapists, when: once they review the eligible criteria, how: review of health records, duration of the recruitment period: 1 months, monitoring recruitment during the trial: home visits and phone calls (Fig. 2).

- **Intervention**: The control group will include patients subject to conventional clinical assessments and in-home oxygen supply control. The intervention group will be subject to conventional follow-up procedures and will use the mobile app “AppO2”. Sampling technique: consecutive. Patients will be recruited as they are captured and will be randomly assigned to a group with the statistical software EPIDAT, 3.1. Masking: single (Participant)
- **Data sources**: Self-registration will be used, which will reveal the potential role of the app in such registry. Moreover, there will be clinical records, in which any significant findings observed at a home visits will be recorded. Users will be surveyed based on the patient’s behavior and the use of in-home oxygen supplies.

#### Eligibility criteria (inclusion and exclusion criteria)

To obtain a homogeneous study population, the following eligibility criteria have been established:

Inclusion criteria.

- Subjects of 18 years and older.
- Subjects with PaO_2_ < 60 mmhg, SO_2_ < 89% and dyspnea. Patients on home oxygen therapy enrolled in a home healthcare program during the study period.
- Subjects whose disease has progressed for 1 year or more.
- Subjects accepting to participate in the study by providing informed consent.

Exclusion criteria.

The following subjects will be excluded from the study:

- Subjects with invasive and/or non-invasive mechanical ventilation.
- Subjects incapable of using the mobile app or subjects without a smartphone with an operating system (Android or iOS) and available mobile data connection.

### Study variables

The following outcome variables and independent variables are suggested to reach the study purpose:

### Outcome variables

Oxygen saturation, measured by a pulse oximeter, as well as capillary refill, respiratory rate, heart rate, and duration of the oxygen cylinder. Will be evaluated up to 6 weeks, from date of randomization until the date of first documented progression or date of death from any cause, whichever came first, assessed up to 6 months

Oxygen saturation/fraction of inspired oxygen ratio. Will be evaluated up to 6 weeks, from date of randomization until the date of first documented progression or date of death from any cause, whichever came first, assessed up to 6 months.

Saint George questionnaire for assessing health-related quality of life (HRQoL)  [30]. This questionnaire is used to measure the effects of respiratory diseases such as COPD and asthma. It comprises 50 items divided into three levels: symptoms, activity, and impact. Scores at the symptoms level describe the frequency and severity of respiratory symptoms. Scores at the activity level assess those activities that are limited by breathlessness. Scores at the impact level estimate the psychological and social changes caused by the disease, and patients answer the questions by themselves or in an interview. Its estimated duration is 10 min. Moreover, scores are individually calculated for each level and globally and range from 0 (no changes in the quality of life) to 100 points (maximal alteration). will be evaluated up to 6 weeks, from date of randomization until the date of first documented progression or date of death from any cause, whichever came first, assessed up to 6 months.

Borg dyspnea scale [31]. It is an analogue, standardized and validated scale written in Spanish, which can be applied quickly and easily to graphically assess the patients and their perception of dyspnea. It has been used since the 70 s and comprises a score ranging from 0 to 10. The scale determines the severity of the respiratory distress and has a graph close to each quantitative value, which helps patients identify their respiratory distress perception: number 10 being the highest level of dyspnea perception. Will be evaluated up to 6 weeks, from date of randomization until the date of first documented progression or date of death from any cause, whichever came first, assessed up to 6 months

Identification of dyspnea self-management and quality of life through the mobile app. Will be evaluated up to 12 weeks, from date of randomization until the date of first documented progression or date of death from any cause, whichever came first, assessed up to 6 months

- Independent variables: medical diagnosis, comorbidities, age, sex, health system affiliation regime, smoking, alcohol use, obesity, social isolation, number of people who live with the patient, caregivers required for the patient’s healthcare, prescription and compliance with oxygen therapy (hours/days), underuse of the oxygen system, level of instruction for the management of the mobile app.

#### Description of the mobile app

The mobile app has four sections: the first one is called “patient” (Picture 1) and comprises two parts: progression and medical record. In the first part, users can upload data regarding their heart rate, respiratory rate, arterial oxygen saturation, capillary refill, dyspnea level, fraction of inspired oxygen, and any relevant observations (additional results). In the second part, patients can see their vital sign trends within the last week, which has been included to simplify the decision-making process regarding the requirement for oxygen therapy. In this sense, it is worth mentioning that AppO_2_ displays a visual alarm if the vital signs uploaded fall outside predetermined limits. Moreover, patients’ data can be deleted once the follow-up period is terminated.

The second section is called “vital signs” (Picture 2) and explains in detail the way in which each vital sign is measured to ensure the highest level of accuracy of input data. Moreover, there is a third section called “formulas” (Picture 3), in which the duration of an oxygen cylinder and the oxygen saturation/fraction of inspired oxygen ratio can be estimated.

### Formulas: duration of an oxygen cylinder.

where P is pressure of the cylinder; RP is residual pressure; F is conversion factor of the cylinder; and Fl is oxygen flow intended to be used.

### SO_2_/FIO_2_ ratio

For a noninvasive saturation monitoring of the patient, the SaO_2_/FiO_2_ (oxygen saturation/fraction of inspired oxygen) formula will be used [32–34]. In this manner, gasometric assays are avoided, and continuous respiratory monitoring can be achieved. Oxygen saturation levels will be recorded, in addition to the fraction of inspired oxygen (FiO_2_) that is determined on the basis of the oxygen system required by the patient. This value will be recorded on a weekly basis. Note that SO_2_ is arterial oxygen saturation and FiO_2_ is the fraction of inspired oxygen.

The fourth section is called “About”. In this section, patients and caregivers will have access to useful information regarding the management of oxygen at home such as relevant safety and hygiene advice regarding oxygen supplies and systems. Moreover, it will have a Frequently Asked Questions section (FAQs) about the management of oxygen at home, which will include the following questions:

- Why do I have to use oxygen at home?
- How do I use my oxygen systems?
- When do I have to use my oxygen systems?
- Will I use oxygen forever?
- Can I increase oxygen liters based on my prescription?
- How can I clean my oxygen supplies and system?
- When can I remove my oxygen supplies?
- Is my health negatively affected by oxygen? (Table 1)

**Table 1:** Items from the World Health Organization Trial Registration Data Set

Data category	Information
Primary registry and trial identifying number	ClinicalTrials.gov NCT04820790
Date of registration in primary registry	March 29, 2021
Secondary identifying numbers	Not Applicable
Source(s) of monetary or material support	Todomed
Primary sponsor	Todomed
Secondary sponsor(s)	Not applicable
Contact for public queries	Anisbed Naranjo Rojas, magister, + 573006658955anisbednaranjo24@gmail.com
Contact for scientific queries	TodoMed, Colombia
Public title	Mobile Application for Monitoring Patients with Home Oxygen. A Protocol of a Randomized and Controlled Clinical Trial
Scientific title	Mobile Application for Monitoring Patients with Home Oxygen. A Protocol of a Randomized and Controlled Clinical Trial
Countries of recruitment	Colombia
Health condition(s) or problem(s) studied	Oxygen-therapy
Intervention(s)	Experimental: Experimental Group 1 The efficiency of the mobile app in the follow-up of patients with home oxygen will be evaluated during 6 months No Intervention: Intervention Group 2: Regular monitoring of the home oxygen without mobile app during 6 months
Key inclusion and exclusion criteria	Inclusion Criteria: Individuals of 18 years of age and older Patient with PaO2 < 60 mmHg, SO2 < 89% and dyspnea. Patients with home oxygen therapy enrolled in-home care programs during the study period. Time of evolution of the disease greater to one year Patients who express their willingness to participate in the study through their informed consent. Exclusion Criteria: Patients with the following exceptional situations will be excluded from the study: Patients with invasive and/or non-invasive mechanical ventilation Patients with the inability to operate a mobile application or those who do not have a smartphone with an operating system (Android or iOS) and available data connection.
Study type	Interventional (Clinical Trial), Allocation: Randomized Intervention Model: Parallel Assignment Masking: Single (Participant) Primary Purpose: Supportive Care
Date of first enrolment	June 1, 2021
Target sample size	44
Recruitment status	Not yet recruiting
Primary outcome(s)	Recognition of self-management of dyspnea and through the mobile app (Time Frame: up to 12 weeks for 6 months)
Key secondary outcomes	Saint George questionnaire for the assessment of health-related quality of life (Time Frame: up to 6 weeks for 6 months)

Criteria for discontinuing or modifying allocated interventions for a given trial participant

Voluntary abandonment, death.

Participant Retention

Once a patient is enrolled or randomized, the study site will use reasonable efforts to follow the patient throughout the study period.

Interaction between users and the AppO_2_ mobile app.

The app has a user-friendly design through which patients can intuitively access the different sections and accordingly register their data. When patients log in for the first time, they have to enter their basic data such as full name, healthcare system affiliation regime, birth date, sex, and diagnosis. Once a patient’s account has been created, the following healthcare-derived clinical results can be recorded in the “progression” section: heart and respiratory rates, SO_2_ measured with a pulse oximeter, level of dyspnea, capillary refill, and fraction of inspired oxygen. After recording a second progression, patients can see a line graph showing trends for each vital sign. Regarding the formulas included, users just have to select the fields of those elements whose values are not predetermined. This ensures that the use of formulas is not limited by a lack of information regarding certain values. In cases with limited and standardized available options (e.g., type of cylinder), there is a drop-down list such as that the desired option can be quickly selected.

### Reports

The mobile app will have a section called “changes”, which will comprise notifications made to users when they register changes in the management and frequency of home oxygen therapy such as reports of changes of systems and oxygen flow. If patients do not upload their management behavior report, the app will send a notification to encourage such upload. Hernández de los Reyes et al. [35] evidenced that the best time to send such remainders can be selected based on a) the best time of the day to receive notifications as determined by users of the app and b) the possibility of sending messages at time points in which users’ routine or sleeping times are not interrupted. For these reasons, we have determined that the best adherence will be achieved at time points in which patients have no commitments (one notification in the morning and one at night, twice a month during the 6 months of the intervention).

### Ethical considerations

Ethical approval and informed consent. The ethical consent approved by the participants will be obtained in writing.

This study protocol complies with the Declaration of Helsinki for medical studies and has been approved by the Ethics and Bioethics Committee of Universidad Santiago de Cali, and has been registered on the Clinicaltrials.gov platform (Identifier: NCT04820790). All patients will receive written communication of the purposes of the study and will sign an informed consent pursuant to current regulations. Patients shall sign the informed consent accepting the study conditions.

• Consent for publication: institutional consent form. Has been submitted through the platform.

• Availability of data and material: not Applicable. As the submitted article is a clinical trial protocol, the authors do not have a database to share in any public repository. Once the clinical trial is completed, the investigators will decide the most appropriate way to make them public.

#### Confidentiality

All study-related information will be stored securely at the study site. All principal investigators will have access to the clean data sets.

If administrative changes are made to the protocol, they will be minor corrections and / or clarifications that will have no effect on the way the study will be conducted. These administrative changes will be documented in a memorandum.

• Conflict of interests: This research was reviewed by the Todomed company and they did not propose changes.

• Funding: this research has been supported by the Todomed company, collection of data. Todomed as a financial institution will support by sharing the necessary facilities to carry out interviews, follow-up consultations, etc. It will also provide all medical care to the study participants and control of biochemical parameters. In other words, Todomed's support consists of providing all care in medical facilities.

#### Statistical analysis

A descriptive analysis of the study variables will be performed, characterizing patients, generating graphs and frequency tables for qualitative variables, and estimating measures of central tendency (mean), dispersion (standard deviation) and position (distribution limits and range) for quantitative variables.

For bivariate analysis, a Student’s t test will be conducted to compare two means, whereas a chi-squared test and Fisher’s exact test will be used for qualitative variables. For analyzing three mean values or more, a repeated measures analysis of variance (ANOVA) will be used. Multiple lineal regression models will be applied to adjust the impact of the use of the app and to remove the effect of confusing factors, estimating standardized beta coefficients, an adjusted determination coefficient, and residual values. An alpha error of < 5% will be accepted and 95% confidence intervals (95% CI) will be estimated for all statistical analysis, and the SPSS Statistics software, 25.0 version (IBM Corp) will be used.

**Formal committee**: no need for a formal committee as the trial is short with minimal known risks.

## Discussion

The general purpose of this protocol is to assess the efficacy of a mobile app in the follow-up of patients under home oxygen therapy through an intervention aimed at decreasing acute exacerbations. Moreover, this protocol intends to determine the suitability of oxygen system prescriptions and to identify oxygen supply underuse at home. This intervention will be assessed through a randomized, two-arm clinical follow-up. Literature suggests that the use of mobile devices to monitor patients’ health or locate patients with chronic diseases or conditions has become possible [36, 37]. Mobile apps can provide public healthcare, improve community data collection, or provide remote assistance for controlling medical data of patients with chronic diseases [38, 39]. Certain studies show that the use of mobile devices can improve decision-making processes and diagnostic suitability and provide immediate access to multiple medical data sources [40–42]. If the results of our clinical trial suggest a positive effect, we will demonstrate that the continuous monitoring of patients on home oxygen therapy, and the creation of a direct link with their caregivers, will result in a decrease in complications and an optimization of home oxygen management.

## Data Availability

not Applicable. As the submitted article is a clinical trial protocol, the authors do not have a database to share it in any public repository. Once the clinical trial is completed, the investigators will decide the most appropriate way to make them public.

## Abbreviations

CHOT: Continuous home oxygen therapy
APP: Applications
ICT: Information and communication technologies
E-HEALTH: Information and communication technologies set
DT: Duration time of the oxygen cylinder
FL: Flow
F: Conversion factor
P: Pressure of the cylinder
RP: Residual pressure
SatO_2_: Saturation of oxygen
FiO_2_: Fraction of inspired oxygen
